# A complex RARE is required for the majority of *Nedd9* embryonic expression

**DOI:** 10.1007/s11248-014-9825-9

**Published:** 2014-08-14

**Authors:** Danielle C. Knutson, Margaret Clagett-Dame

**Affiliations:** Department of Biochemistry, University of Wisconsin-Madison, 433 Babcock Drive, Madison, WI 53706 USA

**Keywords:** All-*trans* retinoic acid (atRA), Retinoic acid response element (RARE), *Nedd9*, *Hef1*, Nervous system development, Cranial nerves

## Abstract

Neural precursor cell expressed, developmentally down-regulated 9 (*Nedd9, Casl, Hef1, p105cas, Ef1*) is a scaffolding protein that assembles complexes involved in regulating cell adhesion, migration, division, and survival. *Nedd9* is found very early in the developing embryonic nervous system. A highly conserved complex retinoic acid response element (RARE) is located 485 base pairs (bp) upstream of exon 2B in the promoter of the *Nedd9* gene. Mice transgenic for a 5.2 kilobase (kb) region of the 2B *Nedd9* promoter containing the RARE upstream of a *lacZ* reporter gene [*Nedd9*(RARE)-*lacZ*] show a large subset of the normal endogenous *Nedd9* expression including that in the caudal hindbrain neuroepithelium, spinal cord, dorsal root ganglia (drg) and migrating neural crest (ncc). However, the transgenic mice do not recapitulate the native *Nedd9* expression pattern in presumptive rhombomeres (pr) 3 and 5 of the early hindbrain, the base of the neuroepithelium in the midbrain, nor the forebrain telencephalon. Thus, the 5.2 kb region containing the intact RARE drives a large subset of *Nedd9* expression, with additional sequences outside of this region needed to define the full complement of expression. When the 5.2 kb construct is modified (eight point mutations) to eliminate responsiveness of the RARE to all-*trans* retinoic acid (atRA) [*Nedd9*(mutRARE)-*lacZ*], virtually all β-galactosidase (β-gal*, lacZ*) expression is lost. Exposure of *Nedd9*(RARE)-*lacZ* transgenic embryos to excess atRA at embryonic day 8.0 (E8.0) leads to rostral ectopic transgene expression within 6 h whereas the *Nedd9*(mutRARE)-*lacZ* mutant does not show this effect. Thus the RARE upstream of the *Nedd9* 2B promoter is necessary for much of the endogenous gene expression during early development as well as ectopic expression in response to atRA.

**Electronic supplementary material:**

The online version of this article (doi:10.1007/s11248-014-9825-9) contains supplementary material, which is available to authorized users.

## Introduction

All-*trans* retinoic acid (atRA) is a metabolite of vitamin A that is essential to support normal growth and differentiation in a wide variety of cells and tissues (Clagett-Dame and DeLuca 2002; Clagett-Dame and Knutson 2011). It regulates many aspects of morphogenesis and organogenesis (Rhinn and Dolle 2012). In the developing nervous system, atRA is required to pattern the posterior hindbrain/anterior spinal cord region, and later, for neuronal differentiation (Clagett-Dame and Knutson 2011; Gavalas and Krumlauf 2000; Glover et al. 2006). atRA acts by binding to nuclear retinoic acid receptors (RAR). These receptors when bound to ligand regulate the transcription of atRA-responsive genes in a cell type and tissue-specific manner. The RAR binds to the retinoid X receptor (RXR), forming a heterodimer that binds with high affinity to DNA sequences called RAREs (Chambon 1996). Formation of the atRA-receptor complex initiates the release of repressor proteins and the recruitment of coactivators to the site, thus opening up the chromatin structure and facilitating transcriptional response (Samarut and Rochette-Egly 2012). Binding of the RAR/RXR complex to these RARE enhancer sequences is the major mechanism whereby atRA directly regulates target gene expression (Balmer and Blomhoff 2002).

The identification of target genes that play a role downstream of atRA and its receptors in nervous system development is an active area of investigation. Our lab identified *Nedd9* (*Casl, Hef1, p105cas, Ef1*) as a gene that is rapidly induced after the exposure of SH-SY5Y human neuroblastoma cells to atRA (Merrill et al. 2004a, b). *Nedd9* is a member of the Crk-associated substrate (Cas) family of multidomain docking or scaffolding proteins that lie downstream of integrin-initiated signal transduction pathways (Tikhmyanova et al. 2010). Cell culture studies suggest that the NEDD9 protein influences multiple biological processes, including regulation of cell shape, cell migration, apoptosis and the cell cycle (Singh et al. 2007). In response to atRA, SH-SY5Y cells differentiate and undergo morphological changes, including the extension of neurites and changes in adhesion (Pahlman et al. 1984). Ectopic overexpression of NEDD9 in PC12 (pheochromocytoma) cells or in MCF-7 (human breast carcinoma) cell lines also treated with a Rho kinase inhibitor leads to the formation of neurite-like membrane extensions (Bargon et al. 2005; Sasaki et al. 2005) supporting the idea that NEDD9 might participate in mediating the effects of retinoid on neurite outgrowth and/or changes in cell adhesion properties.

*Nedd9* is expressed in the nervous system of the developing embryo, including the early hindbrain and spinal cord. Previously, our group showed that *Nedd9* mRNA is expressed very early in the node, presumptive rhombomeres of the developing hindbrain, neuroepithelium and floor plate, spinal cord, dorsal root and cranial ganglia, and a variety of other tissues including the otocyst, pharyngeal arches, lung, foregut, kidney and cartilage primordia, and early vasculature (Merrill et al. 2004a, b). Another group showed that *Nedd9* is also expressed in ventral midbrain, diencephalon, progenitors of pontine and cerebellar nuclei, dorsal motor nucleus of the vagus nerve, the hypoglossal nucleus and in non-lineage-restricted neural crest progenitor cells (Aquino et al. 2008, 2009). However, it is not known whether *Nedd9* gene expression in any of these regions is under the direct regulation of atRA and its receptors.

A 31-bp atRA RARE is present approximately 450 bps 5′ of the transcriptional start site of the 2B promoter of the *Nedd9* gene (Knutson and Clagett-Dame 2008; Merrill et al. 2004b). The *Nedd9* RARE is more complex in structure than many RAREs that have been reported. Classical RAREs are composed of two direct repeats of PuG(G/T)TCA separated by a variable number of bases; 5, 2, or 1 (Chambon 1996; Mangelsdorf 1994), whereas binding elements with more diverse spacing and topology have also been identified (Al Tanoury et al. 2013). The *Nedd9* RARE is composed of four direct repeats separated by 1, 5 and 1 bps, all of which are needed for maximum induction of a transiently transfected reporter by atRA. Regulation of *Nedd9* gene transcription via the direct binding of the RAR/RXR complex to this RARE enhancer in cultured cells was previously confirmed using ChIP analysis (Knutson and Clagett-Dame 2008).

In the present study, transgenic reporter mice containing approximately 5.2 kb of the *Nedd9* 2B promoter with an intact RARE or with the RARE rendered nonfunctional by eight point mutations were generated and studied. The contribution of the RARE to the endogenous *Nedd9* spacio-temporal expression pattern is examined, and the importance of the intact RARE in mediating response to exogenously administered atRA is explored.

## Materials and methods

### *Nedd9* β-galactosidase transgene constructs

The *Nedd9* β-gal transgene constructs were generated using the plasmid pUC19/AUGβgal (obtained from Dr. Eric N. Olson, UT Southwestern) (Cheng et al. 1992). The pUC19/AUGβgal plasmid contains an alcohol dehydrogenase translational start codon and a Simian Virus 40 polyadenylation sequence located at the 3′ end of the *lacZ* coding sequence. The plasmid also contains the pCMVβ Simian Virus 40 splice donor/splice acceptor cassette (Clontech) 5′ of the *lacZ* coding sequence in order to increase *lacZ* expression (Choi et al. 1991). The splice cassette, which was generated by BspEI and XmaI restriction digest of the plasmid pCMVβ (Clontech), was ligated into a unique XmaI restriction site in the pUC19/AUGβgal vector prior to the addition of the *Nedd9* promotor region.

The intact RARE *Nedd9* plasmid (Online Resource 1a–c), “*Nedd9*(RARE)-*lacZ*” was constructed by amplifying a 5.4 kb region (−5,366, +15) from the human bacterial artificial chromosome (Online Resource 1b; BAC; RP11-263D22, CHORI; Oakland, Ca) with the upstream primer 5′-TAG TCC CAC GAG GGT GGA-3′ and the downstream primer 5′-ATC GTG TCG ACC GAA CTC CGG GAA CAA AA-3′, which includes an internal SalI site that reduces the fragment to 5.2 kb. The amplified DNA was cloned into the pGEM-T Easy Vector (Promega), and a positive clone was verified by restriction digest followed by sequence analysis. Next, an SphI and SalI fragment (−5,177, +15) was subcloned into pUC19/AUGβgal plasmid cut at unique SphI and SalI sites (Online Resource 1c). A positive clone was verified first by restriction digest followed by sequence analysis. The modified RARE *Nedd9* plasmid, “*Nedd9*(mutRARE)-*lacZ*”, was constructed by amplifying a fragment containing the RARE with eight point mutations (−842, +15) from the hM-1/2/3/4 plasmid described previously (Knutson and Clagett-Dame 2008) with the upstream primer 5′-AGA CGC GAA GGG AAT CAG A-3′ and the downstream primer 5′-ATC GTG TCG ACC GAA CTC CGG GAA CAA AA-3′ (which also includes an internal SalI restriction site), and cloning it into the pGEM-T Easy Vector. A positive clone was verified first by restriction digest followed by sequence analysis. A BstEII and SalI fragment (−842, +15) containing the mutant RARE was then used to replace this same region in the *Nedd9*(RARE)-*lacZ* plasmid. A positive clone was verified first by restriction digest followed by sequence analysis.

Prior to use for pronuclear microinjection, plasmids were tested for atRA-regulated expression in a cell culture system. MCF-7 cells were transfected and assayed as previously described (Knutson and Clagett-Dame 2008). Briefly, cells were seeded in six-well plates and were transfected in triplicate with reporter plasmid, pGL3-Basic (internal luciferase control), and RAR and RXR expression plasmids using FuGENE 6 and evaluated for both β-gal and luciferase activity. β-gal values are normalized to luciferase values. Data shown is the average of triplicate wells ± the standard error (Online Resource 1d).

### Production and study of transgenic animals

Transgenic mice were produced by pronuclear microinjection of the intact RARE construct [*Nedd9*(RARE)-*lacZ*] or modified RARE construct [*Nedd9*(mutRARE)-*lacZ*] using NarI and SphI linearized fragments according to established methodologies by the Roswell Park Cancer Institute (RPCI) Gene Targeting and Transgenic Core Facility. Transgenic founders were sent to the University of Wisconsin-Madison. All animal studies were performed under an approved Animal Care and Use Committee (ACUC) animal protocol according to institutional guidelines at the University of Wisconsin-Madison. Treatment of mice was in accordance with policies of the Institutional Animal Care and Use Committees (IACUC) at both institutions.

Founder animals for each transgene were identified by PCR genotyping of DNA isolated from tail biopsies (Online Resource 1e). For the intact RARE construct, *Nedd9*(RARE)-*lacZ,* two independent founder lines, wt4966 and wt5000, were successfully established. Both the level of β-gal staining intensity as well as the spatiotemporal expression pattern appeared similar for the wt4966 and wt5000 lines. Injection of the modified RARE resulted in five independent founder lines (mut5879, mut6636, mut6644, mut6646 and mut6653), three of which were extensively characterized. Both the level of β-gal staining intensity as well as the spatiotemporal expression pattern appeared similar for these three mutant lines (mut5879, mut6646, and mut6653). The staining pattern of a fourth line (mut6644) was also similar, but was not included in the results as we were unable to establish this line before the founder died. A fifth mutant line was discontinued as the staining differed from the other four lines, likely the result of positional effects.

### Embryo generation

The majority of transgenic embryos were generated by mating male transgenic mice with female wild-type (WT) (C57Bl/10Ros-*p* ^*d*^ × C3h/HeRos) mice (389 timed matings) with a lesser number resulting from male WT and female transgenic crosses (86 timed matings). In all crosses, the staining pattern of the transgenic embryos was similar for that construct at each stage examined. Embryos were assigned embryonic day numbers with noon on the day of vaginal plug detection designated as embryonic day 0.5 (E0.5), 4–6 somites (E8.25), 7–11 somites (E8.5) and 12–15 somites (E8.75).

### β-galactosidase staining of mouse embryos to analyze transgene expression

Embryos were fixed at room temperature (rt) in 4 % paraformaldehyde for the following times; E8–9.5 (5 min), E10.5 (15 min), E11.5 (20 min) and E12.5 (30 min). Embryos were then washed at rt twice in PBS (pH 7.2) and then rinse buffer (5 mM EGTA, 2 mM MgCl_2_, 0.01 % Na deoxycholate, and 0.02 % NP-40 in PBS) for 5 to 10 min each. Embryos were stained (5 mM K_3_Fe(CN)_6_, 5 mM K_4_Fe(CN)_6_, 5 mM EGTA, 2 mM MgCl_2_, 0.01 % Na deoxycholate, 1 mg/ml X-gal, 0.02 % NP-40 in PBS) in the dark at 37 °C overnight (~16 h). After washing in PBS for 10 min at rt and post-fixation in 4 % paraformaldehyde overnight at 4 °C, embryos were washed again in PBS at rt for 10 min, and stored in PBS at 4 °C followed by photography using a Nikon model SMZ-U dissection microscope fitted with a 1× lens using a SPOT digital camera (Spotcam, Diagnostic Instruments Inc., Sterling Heights, MI) or Qimaging camera (Qimaging, Surrey, British Colombia) and MetaMorph software (Molecular Devices, Downington, PA). At each stage a minimum of three embryos from five independent litters were analyzed for each of two *Nedd9*(RARE)-*lacZ* lines and three *Nedd9*(mutRARE)-*lacZ* lines.

### In situ hybridization

A 841-bp mouse *Nedd9* probe was amplified using mRNA isolated from adult mouse lung using the upstream primer 5′-ACC GCG GTG GAC AAA GTA GAG C-3′ and the downstream primer 5′-AGA GGG CGT CGA TGG CGT TGA GTA G-3′ (1,628-2,468 based on the mouse *Nedd9* sequence; accession no. NM_017464) and subcloned into pGEM-T Easy Vector (Promega). The sequence was 99 % identical to NM_017464 and differed at 3 non-sequential base pairs (1,756 A to G, 1,996 C to T, and 2,087 T to C). Whole-mount in situ hybridization was carried out using embryos fixed in 4 % paraformaldehyde by the methods previously described (Stern 1998; White et al. 2000; Wilkinson 1998) with the following modifications. The following proteinase K (pK)/refix times were used at each stage; E8.5 (12/10 min), E9.5 (12/10 min), E10.5 (20/10 min) for embryos in whole-mount, and at E11.5 (10/8 min) for vibratome sections (200 μm). Both anti-sense and sense probes were tested. Embryos were imaged as described above.

### Whole-mount immunohistochemistry for Krox20

Whole-mount immunohistochemistry was performed following β-gal staining using previously described methods (McNeill et al. 2010; Wall et al. 1992). Briefly, embryos pretreated with H_2_O_2_ to quench endogenous peroxidase activity were incubated with Krox20 antibody (Covance, Princeton, NJ, USA) diluted 1:500 in TBST (10 mM Tris, pH 8.0, 150 mM NaCl, 0.05 % Tween 20). This was followed by incubation with anti-mouse or anti-rabbit IgG conjugated to horseradish peroxidase (Southern Biotechnology Associates, Birmingham, AL, USA) diluted 1:500 in TBST. Embryos were pre-incubated in a solution of PBT (PBS containing 0.05 % Tween20) containing 0.6 mg/ml 3,3′-diaminobenzidine (DAB; Sigma, St Louis, MO, USA) with 0.75 % NiCl_2_ in PBT followed by the addition of H_2_O_2_ to a final concentration of 0.03 % for color development. Krox20-stained embryos were photographed in PBS as described above.

### Retinoids and treatment of embryos with excess atRA

All-*trans* retinoic acid was purchased from Spectrum Chemical Company (New Brunswick, USA) and was deemed greater than 99 % pure by reverse-phase high-performance liquid chromatography (Motto et al. 1989). Female mice were given a single oral bolus dose of atRA (50 mg/kg) in soybean oil or vehicle at E8.0, and were euthanized 6 h later.

## Results

### Generation of the *Nedd9* RARE-*lacZ* reporter mice

Transgenic reporter mice were generated and included two independent founder lines containing the intact unmodified *Nedd9* RARE sequence [*Nedd9*(RARE)-*lacZ*] as well as founder lines in which the RARE was rendered unresponsive to atRA by mutation [*Nedd9*(mutRARE)-*lacZ*] of which three lines were extensively characterized. Using a transfection cell reporter assay we previously showed that a fragment of the *Nedd9* 2B 5′ promoter sequence (−2,756 to +15) containing the complex RARE is responsive to atRA (Knutson and Clagett-Dame 2008). In designing the *Nedd9* transgenic targeting constructs, the region was extended to −5,177 to +15 bp in order to encompass any additional nearby sites that might be important for transcriptional regulation in vivo (Online Resource 1). Additional mice with the RARE region mutated at eight positions were generated to determine the contribution of the *Nedd9* RARE to its spatiotemporal expression pattern. Prior to injection into fertilized oocytes, each of the targeting constructs was tested for both β-gal activity as well as atRA induction in a transient transfection assay (Online Resource 1d). The intact and mutant RARE constructs showed similar basal levels of β-gal activity. However, only the cells transfected with the construct containing the intact RARE, *Nedd9*(RARE)-*lacZ*, showed an increase in expression upon addition of atRA, with no increase observed in atRA-treated cells transfected with the mutated element, *Nedd9*(mutRARE)-*lacZ*.

### The* Nedd9*(RARE)-*lacZ* transgene containing the intact RARE recapitulates a large subset of the endogenous* Nedd9* mRNA expression pattern

**Fig. 1:**
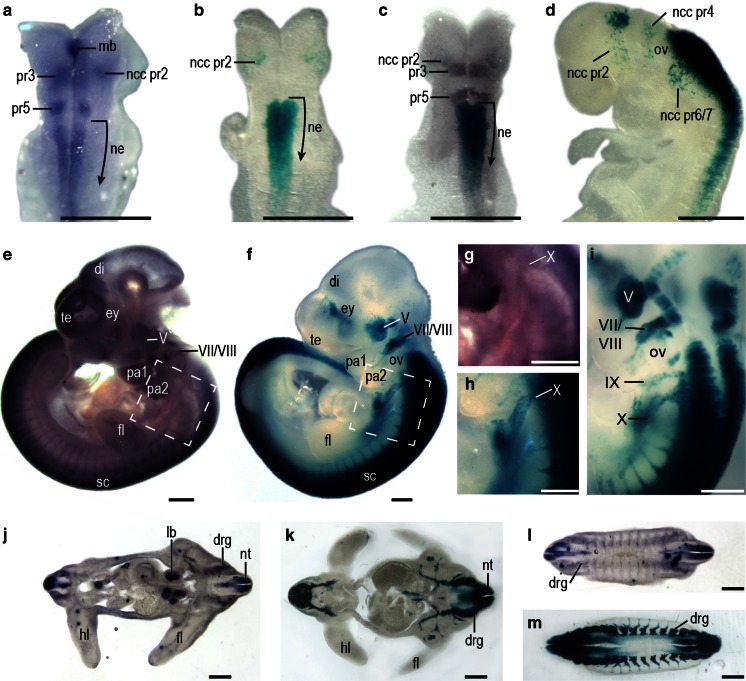
Comparison of endogenous and transgenic *Nedd9* expression (**a**–**c**) *Nedd9* expression at E8.5. Endogenous *Nedd9* expression in a representative WT embryo as determined by in situ hybridization (**a**) is compared to β-gal stained *Nedd9*(RARE)-*lacZ* in a transgenic embryo (**b**). The *lacZ* expression pattern in two independent transgenic lines was similar (data not shown). An antibody to Krox20 (*brown stain*) was used to confirm the location of pr3 and pr5. Analysis of a β-gal stained *Nedd9*(RARE)-*lacZ* embryo before (**b**) and after (**c**) immunohistochemical analysis using an antibody to Krox20. (**d**) *Nedd9* transgene expression at E8.75. **e**–**i** *Nedd9* expression at E10.5. Endogenous *Nedd9* expression in a WT embryo as determined by in situ hybridization (**e**) compared to a β-gal stained *Nedd9*(RARE)-*lacZ* transgenic embryo (**f**); the region of cranial nerve X is boxed, and enlarged for each embryo, respectively, in (**g**) and (**h**). *Nedd9* transgene expression in cranial nerves at E10.5 (**i**). Sections from a WT E11.5 embryo that was vibratome sectioned (200 µm) prior to in situ hybridization show endogenous *Nedd9* expression (**j**, **l**). Vibratome sections (150 µm) from a E11.5 β-gal stained *Nedd9*(RARE)-*lacZ* transgenic embryo (**k**, **m**). *Scale bar* is 50 µm. *Abbreviations* *mb* midbrain, *ne* neuroepithelium, *pr3* presumptive rhomobomere 3, *pr5* presumptive rhomobomere 5, *ncc pr2* neural crest cells migrating from presumptive rhomobomere 2, *ncc pr4* neural crest cells migrating from presumptive rhomobomere 4, *ncc pr6/7* neural crest cells migrating from presumptive rhomobomere 6/7, *ov* otic vesicle, *te* telencephalon, *di* diencephalon, *ey* eye, *V* trigeminal nerve, *VII/VIII* facio-acoustic nerve, *IX* glossopharyngeal nerve, *X* vagal nerve, *pa1* pharyngeal arch 1, *pa2* pharyngeal arch 2, *sc* spinal cord, *drg* dorsal root ganglia, *nt* neural tube, *hl* hindlimb, *fl* forelimb, *lb* lung bud

*Nedd9* expression was examined in WT embryos by in situ hybridization using a riboprobe designed to detect all mRNA variants. At E8.5, WT embryos showed staining in pr3, pr5 and in neural crest cells (ncc) exiting pr2 of the hindbrain (Fig. 1a). *Nedd9* mRNA expression was also observed in the caudal neuroepithelium as well as in the midbrain region (Fig. 1a). In *Nedd9*(RARE)-*lacZ* embryos β-gal staining at E8.5 was observed caudal to pr5 in the hindbrain, with expression extending to approximately somite 7/8 of the neuroepithelium (Fig. 1b, c). Transgene expression was also observed in ncc exiting the hindbrain at the level of pr2 in *Nedd9*(RARE)-*lacZ* embryos (Fig. 1b) and was apparent in ncc exiting pr2, pr4, and pr6/7 at later stages (Fig. 1d). Unlike the WT embryo, staining in pr3 and pr5, as well as the midbrain was absent in *Nedd9*(RARE)-*lacZ* embryos (Fig. 1b).

*Nedd9* mRNA expression was observed in the telencephalon of a WT embryo at E10.5 with lighter staining in the diencephalon. Staining in these forebrain regions was not evident in *Nedd9*(RARE)-*lacZ* embryos (compare Fig. 1e to 1f). However, in both WT and *Nedd9*(RARE)-*lacZ* embryos, staining was observed in the trigeminal (V), facio-acoustic (VII/VIII), and vagal (X) ganglia (compare Fig. 1e and 1f; and Fig. 1g and 1h), and β-gal staining was evident at E10.5 in the cranial nerves of VII/VIII, IX and X (Fig. 1i). Expression was also observed in the neural tube in both WT and *Nedd9*(RARE)-*lacZ* embryos at this stage, and staining in both extended into the tail. Vibratome sections from WT (Fig. 1j, l) and *Nedd9*(RARE)-*lacZ* (Fig. 1k, m) embryos at E11.5 showed staining in a number of structures of neural origin including the neural tube and drg. The *Nedd9*(RARE)-*lacZ* embryos also showed staining in nerves innervating the limbs. However, *Nedd9* expression in the WT lung bud was not recapitulated in *Nedd9*(RARE)-*lacZ* embryos. Thus, the 5.2 kb region immediately upstream of the *Nedd9* promoter containing the RARE drives a large, but not complete, subset of the native gene expression.

### Effect of RARE mutation on embryonic transgenic expression

**Fig. 2:**
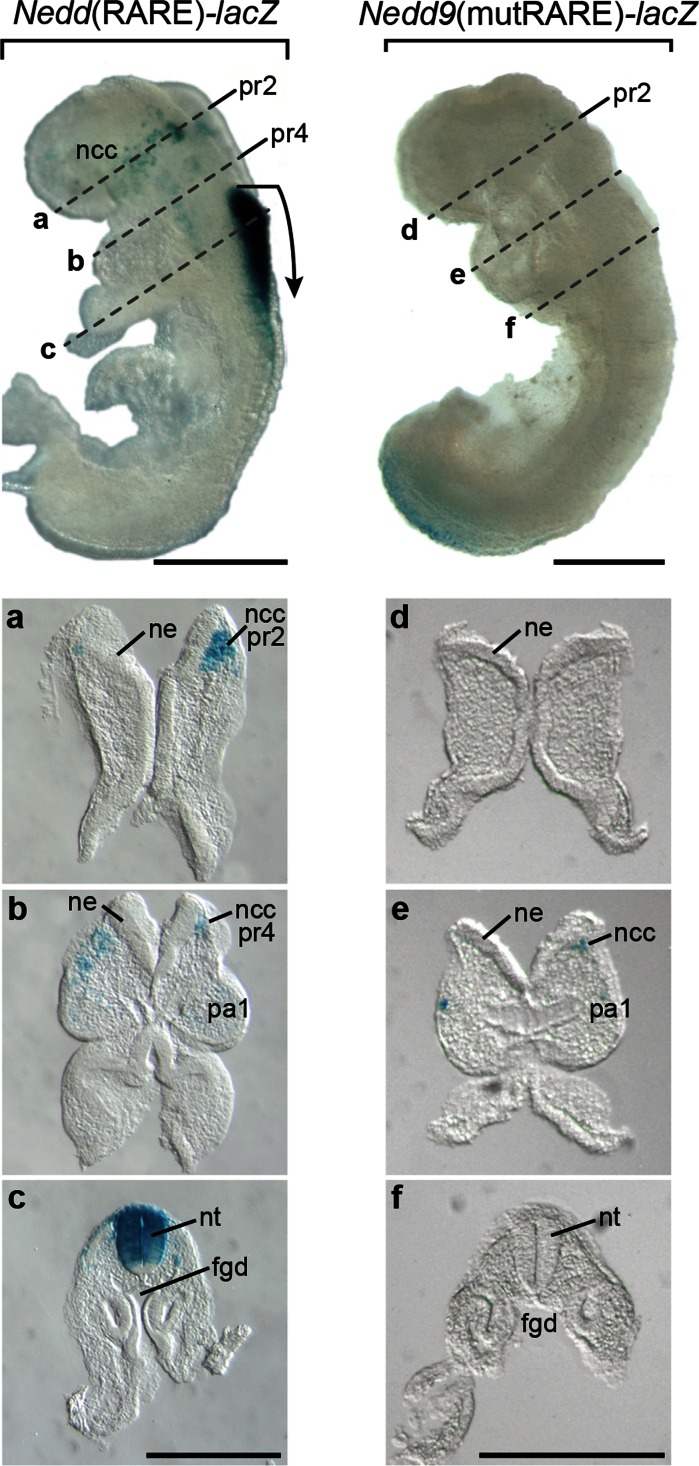
Characterization of the effect of RARE mutation on transgene expression at E8.5. A representative E8.5 *Nedd9*(RARE)-*lacZ* β-gal stained embryo in whole-mount (*left*), and in vibratome sections (**a**–**c**, 60 µm thick). A representative E8.5 *Nedd9*(mutRARE)-*lacZ* β-gal stained embryo in whole-mount (*right*), and in vibratome sections (**d**–**f**, 60 µm thick). *Dotted line* over the embryo indicates the approximate location of the correspondingly labeled section below. *Scale bar* is 50 µm. The *lacZ* expression pattern in all three independent mutant lines was similar (data not shown). The *bent arrow* marks the most rostral site of neuroepithelium expression. *Abbreviations* *ncc* neural crest cells, *pr2* presumptive rhomobomere 2, *pr4* presumptive rhomobomere 4, *ncc pr2* neural crest cells migrating from presumptive rhomobomere 2, *ncc pr4* neural crest cells migrating from presumptive rhomobomere 4, *pa1* pharyngeal arch 1, *ne* neuroepithelium, *nt* neural tube, *fgd* foregut diverticulum

In order to understand the contribution of the *Nedd9* RARE to overall *Nedd9* expression, transgenic lines were generated in which the RARE was mutated making it unresponsive to induction by atRA and its receptors (Knutson and Clagett-Dame 2008). Four lines from independent founders showed similar embryonic staining patterns and three of these lines were extensively studied (Online Resource 1c). At E8.5–9, mutation of the RARE resulted in a complete loss of stain in the hindbrain and neural tube in all *Nedd9*(mutRARE)-*lacZ* transgenic lines (Fig. 2). In addition, the majority of *lacZ* expression in cells that had begun migrating from the lateral edges of the anterior hindbrain (ncc) adjacent to pr2 and pr4 at this time was also absent in the mutants. Loss of *lacZ* expression in ncc and neural tube of the mutant was also confirmed in vibratome sections (compare Fig. 2a–c with Fig. 2d–f).

**Fig. 3:**
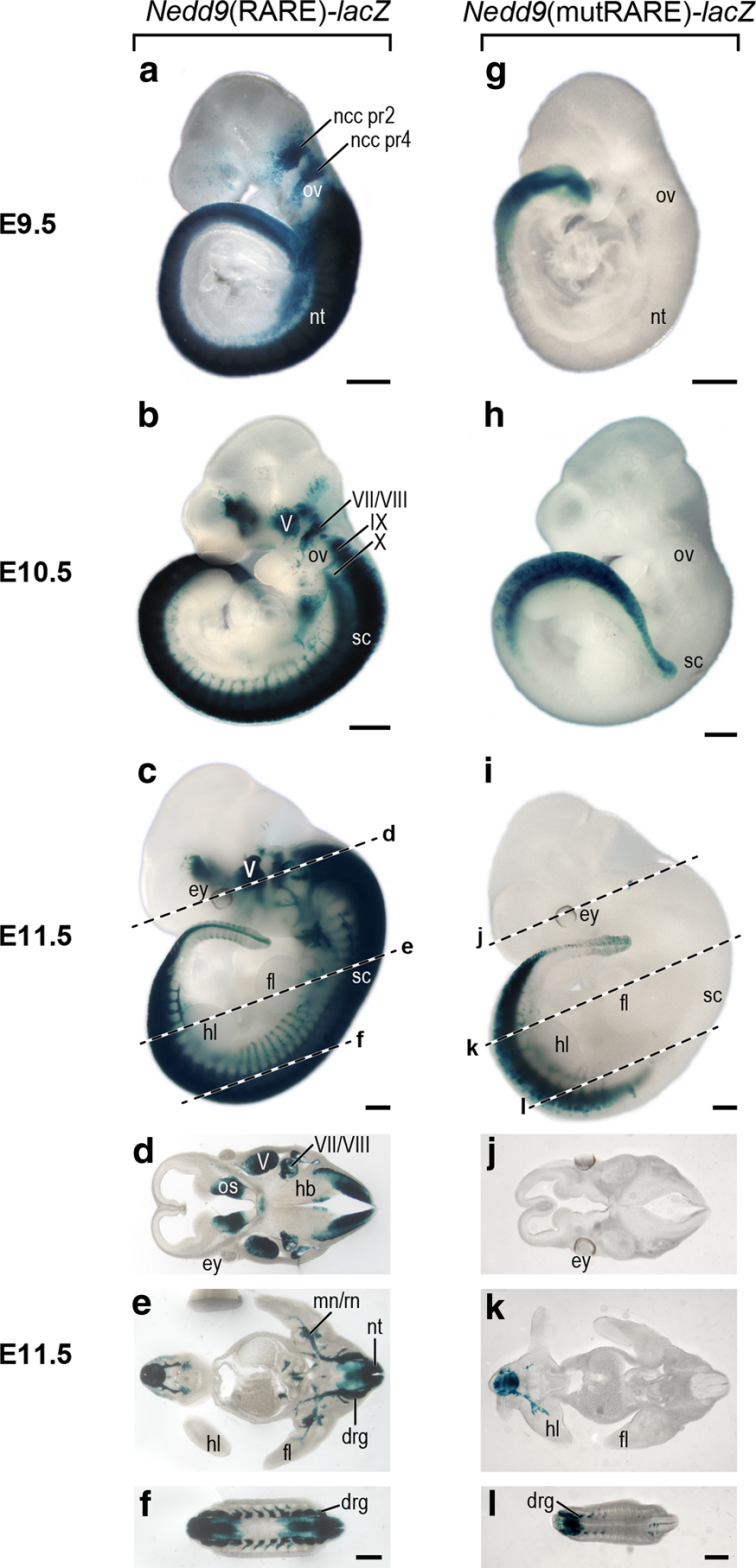
Characterization of the effect of RARE mutation on transgene expression at E9.5–E11.5 (**a**–**f**) *Nedd9*(RARE)-*lacZ* β-gal and (**g**–**l**) *Nedd9*(mutRARE)-*lacZ* β-gal stained embryos. Representative embryos at E9.5 (**a**, **g**), E10.5 (**b**, **h**), and E11.5 (**c**, **i**) are shown. Vibratome sections (150 µm) of the E11.5 embryo shown in (**c**, **i**); *dotted lines* over the embryo indicate the approximate location of the sections (**d**–**l**). *Scale bar* is 50 µm. *Abbreviations* *ncc pr2* neural crest cells migrating from presumptive rhomobomere 2, *ncc pr4* neural crest cells migrating from presumptive rhomobomere 4, *ov* otic vesicle, *V* trigeminal nerve, *VII/VIII* facio-acoustic nerve, *IX* glossopharyngeal nerve, *X* vagal nerve, *ey* eye, *hl* hindlimb, *fl* forelimb, sc spinal cord, *os* otic sulcus, *mn/rn* medial and radial nerves, *hb* hindbrain, *drg* dorsal root ganglia, *nt* neural tube

One day later (E9.5) the neural tube staining as well as staining in the migrating ncc population was absent in the *Nedd9*(mutRARE)-*lacZ* lines, whereas both of these regions were darkly stained in the *Nedd9*(RARE)-*lacZ* transgenics (Fig. 3a vs 3 g). Only staining in the most caudal tail region was observed in the mutant (Fig. 3g). The majority of staining including that in the spinal cord and the developing ganglia of V, VII/VIII, IX and X, as well as in the developing spinal nerves in *Nedd9*(RARE)-*lacZ* embryos at both E10.5 and 11.5 (Fig. 3b, c) was absent in the *Nedd9*(mutRARE)-*lacZ* lines (Fig. 3h, i). *Nedd9*(mutRARE)-*lacZ* lines was confined to the caudal tail region.

To examine transgene expression internally, *Nedd9*(RARE)-*lacZ* and *Nedd9*(mutRARE)-*lacZ* embryos were sectioned on a vibratome. A section through the head at the level of the eye in a E11.5 *Nedd9*(RARE)-*lacZ* transgenic embryo showed strong staining in the optic stalk region, as well as in the ganglia of V, VII/VIII and in the hindbrain (Fig. 3d). The *Nedd9*(mutRARE)-*lacZ* transgenics were devoid of stain in these areas (Fig. 3j). At the level of the limbs, the *Nedd9*(RARE)-*lacZ* transgenic showed *lacZ* expression in a number of structures of neural origin including the entire length of the neural tube, all drg, as well as the nerve tracts extending from the spinal cord including the radial and medial nerves of the both the fore- and hind-limb (Fig. 3c, e). In the *Nedd9*(mutRARE)-*lacZ* transgenics, neural tube and drg expression was only observed in the last 1/3 to 2/3 of the embryo (Fig. 3k, l) and was never as robust as in the *Nedd9*(RARE)-*lacZ* line.

### Expression of the transgenic reporter in* Nedd9*(RARE)-*lacZ* embryos is responsive to atRA and is dependent on an intact RARE

**Fig. 4:**
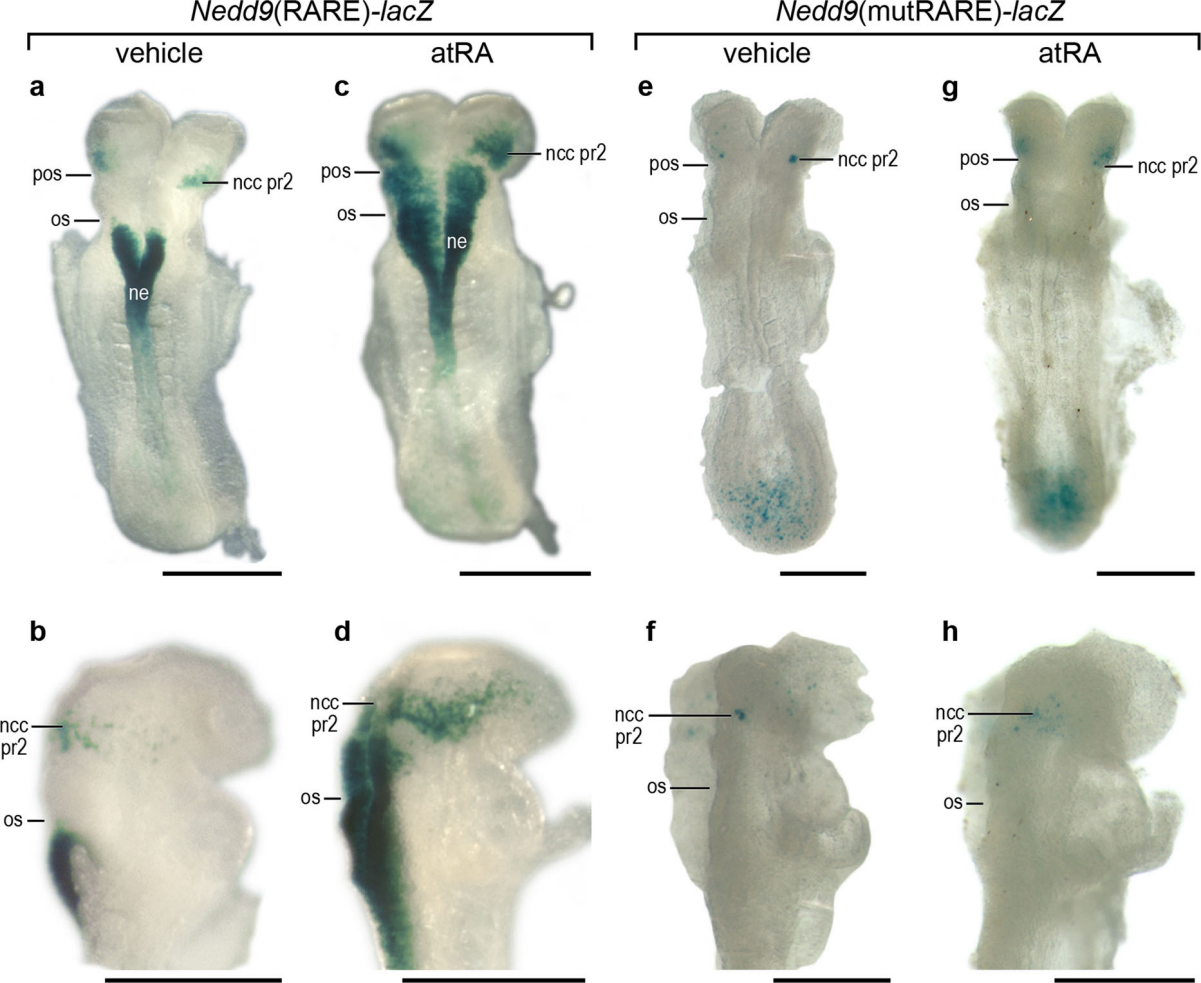
*Nedd9*(RARE)-*lacZ* and *Nedd9*(mutRARE)-*lacZ* embryos exposed to atRA for 6 h. Pregnant dams were dosed at E8.25 with either vehicle (**a**, **b**, **e**, **f**) or atRA (**c**, **d**, **g**, **h**) 6 h prior to embryo collection followed by staining for β-gal activity. Embryos (**a**–**d**) are from a *Nedd9*(RARE)-*lacZ* mating and embyos (**e**–**h**) are from a *Nedd9*(mutRARE)-*lacZ* mating. *Scale bar* is 50 µm. *Abbreviations* *pos* pre-otic sulcus, *os* otic sulcus, *ncc pr2* neural crest cells migrating from presumptive rhomobomere 2, *ne* neuroepithelium

All-*trans* retinoic acid signaling in the developing nervous system is required for normal hindbrain patterning, and this region is also adversely affected by excess exogenous retinoid (Clagett-Dame and DeLuca 2002). In order to determine whether the *Nedd9*(RARE)-*lacZ* or *Nedd9*(mutRARE)-*lacZ* construct respond to atRA in vivo, pregnant mice were given a single oral dose of atRA or control vehicle at E8.25, and the expression of the *Nedd9* transgene was examined 6 h later. The vehicle treated embryos all showed the expected transgene expression pattern for that line (Fig. 4a, b, e, f). At the eight somite stage, vehicle-treated *Nedd9*(RARE)-*lacZ* embryos expressed the transgene in the neuroepithelium adjacent to the somites, starting at the level of the otic sulcus. Additional staining in the mesenchyme was noted just rostral of the preotic sulcus that marks the border between pr2 and pr3, and is consistent with transgene expression in ncc that have just begun to delaminate from the lateral edges of the neural plate (Fig. 4a, b). In *Nedd9*(RARE)-*lacZ* embryos exposed to atRA, there was ectopic transgene expression throughout the anterior hindbrain neuroepithelium caudal to the pr2/3 border, as well as an increase in the number of cells positive for stain migrating into the head region from pr2 (Fig. 4c, d). As expected, embryos carrying the mutant RARE and treated with vehicle lacked staining in the neuroepithelium and in ncc exiting pr2 (Fig. 4e, f), and *Nedd9*(mutRARE)-*lacZ* embryos exposed to atRA at E8.25 showed little additional expression compared to the mutant vehicle control embryos (Fig. 4g, h).

## Discussion

Transgenic mice with 5.2 kb of the *Nedd9* promoter containing a complex RARE and driving *lacZ* expression [*Nedd9*(RARE)-*lacZ* mice] show a distribution pattern overlapping that of the native *Nedd9* mRNA in WT mice. Both endogenous *Nedd9* mRNA and *lacZ* expression in *Nedd9*(RARE)-*lacZ* transgenic mice are observed in the hindbrain epithelium caudal to pr5. At later stages, endogenous *Nedd9* mRNA as well as the expression of the intact *Nedd9*(RARE)-*lacZ* reporter are observed in the dorsal spinal cord, drg, and cranial nerves V, VII/VIII and X. At early stages, *Nedd9*(RARE)-*lacZ* staining is also observed in ncc emanating at all levels of the hindbrain. This is consistent with the previously reported expression of *Nedd9* in non-lineage-restricted neural crest progenitor cells (Aquino et al. 2009).

The loss of the majority of *lacZ* expression in multiple independent transgenic lines with the same 5.2 kb region of the *Nedd9* 2B promoter in which the RARE sequence is mutated at eight point mutations [*Nedd9*(mutRARE)-*lacZ*] reveals the importance of this element in mediating NEDD9 expression in vivo. Earlier work from our group in cell culture showed that mutation of the RARE in this fashion eliminates the response of a reporter gene to atRA (Knutson and Clagett-Dame 2008). The complete or nearly complete loss of reporter expression in transgenic mice carrying the mutant RARE argues that endogenous atRA plays an important role in regulating the expression of *Nedd9*.

The *Nedd9* gene spans nearly 204 kb, and, the *Nedd9* gene has two distinct transcriptional and translational start sites. The two independent promoter regions are depicted in Online Resource 1a. The *Nedd9*(RARE)-*lacZ* construct contains a region that lies directly upstream of the 2B promoter region, shown previously to be regulated by atRA (Knutson and Clagett-Dame 2008). There are regions where *Nedd9* mRNA but not β-gal activity is observed including pr3 and pr5 of the hindbrain, the telencephalon, diencephalon, and ventral midbrain. Thus, regulatory sequences outside of the 5.2 kb transgenic construct are also required for normal *Nedd9* expression, and it is possible these could represent expression regulated through the alternate upstream promoter. However, the present work clearly shows that a majority of *Nedd9* expression in early embryos is regulated through the 2B promoter.

Many regions where *Nedd9*(RARE)-*lacZ* transgene expression is dependent upon the intact RARE are also sites where endogenous atRA activity is present as assessed using embryos carrying a *lacZ* reporter transgene containing multiple copies of the RARβ RARE (β-RARE-*lacZ*). β-RARE-*lacZ* expression is found in the neural plate at the early somite stage caudal to the preotic sulcus, and by the 8–10 somite stage, it is found caudal to the otic vesicle extending to the last formed somite, but is absent or very low in the tail bud, the most posterior region of the embryo (McCaffery et al. 1999; Molotkova et al. 2005; Rossant et al. 1991; Wagner et al. 2000). By E10.5, the expression of the β-*RARE*-*lacZ* transgene in the trunk is strong in the region of the dorsal spinal cord and developing ventral motor axons (Mic et al. 2002; Rossant et al. 1991) both regions where the *Nedd9*(RARE)-*lacZ* transgene is expressed. Much of this β-RARE-driven *lacZ* signal can be accounted for by activity of RALDH2, an enzyme responsible for the final step in atRA synthesis during the early stages of embryonic development (Kumar et al. 2012; Mic et al. 2002). At the early somite stage, RALDH2 is expressed in somitic mesoderm and anterior presomitic mesoderm (Niederreither et al. 1997); the resulting atRA then diffuses to tissues including the neural plate, and at later stages, to the neural tube, with β-RARE-driven *lacZ* signal showing a more rostral border than that of RALDH2 (Molotkova et al. 2005; Sirbu and Duester 2006; Smith et al. 2001). Deletion of the enzymes responsible for catabolizing atRA (CYP26A1 and CYP26C1) results in defects in the migrating ncc population supporting the notion that at least some atRA diffuses into the hindbrain region (Uehara et al. 2007). RALDH3 also contributes to the production of atRA in embryos at later stages, including the developing eye and forebrain (Smith et al. 2001). It is possible that the lack of overlap in *Nedd9*(RARE)-*lacZ* expression and atRA activity (as assessed by the β-RARE-*lacZ* transgene) in some regions of the developing nervous system, including the ncc emanating from the hindbrain and the drg, and later in the optic stalk is due to activation of the *Nedd9* transgene at lower concentrations than needed for that of the β-RARE transgene, or alternatively, the β-RARE-*lacZ* could be silenced in these regions.

*Nedd9*(RARE)-*lacZ* transgenic and *Nedd9*(mutRARE)-*lacZ* transgenic embryos differ in their response to exogenous atRA treatment. While treatment of the *Nedd9*(RARE)-*lacZ* embryos results in a dramatic expansion of expression, no difference in expression is observed between vehicle and atRA treatment for any of the *Nedd9*(mutRARE)-*lacZ* lines indicating a requirement for this RARE to respond to either endogenous or exogenous sources of atRA.

The present work supports the conclusion that a complex RARE contained within a lacZ transgene comprised of a 5.2 kb region of the 2B *Nedd9* promoter is responsible for driving a large subset of the endogenous *Nedd9* expression pattern including that in the hindbrain neuroepithelium caudal to the r5 border, spinal cord, drg and migrating neural crest. However, the transgene does not copy the native *Nedd9* expression in pr3 and pr5 of the early hindbrain, nor the forebrain telencephalon, indicating that gene regulatory sequences outside of the 5.2 kb segment are needed to drive the full complement of *Nedd9* expression. Exposure of *Nedd9*(RARE)-*lacZ* transgenic embryos to excess atRA at E8.25 leads to rostral ectopic transgene expression within 6 h, confirming the atRA-responsiveness of the RARE-containing construct in vivo. In transgenic embryos where the RARE has been mutated the majority of *lacZ* expression is lost. This is observed at every stage analyzed, indicating that atRA signaling through the *Nedd9* RARE is necessary for a significant proportion of endogenous gene expression during development as well as for modification of expression by atRA.

## Electronic supplementary material

Below is the link to the electronic supplementary material.

**Online Resource 1 Strategy for generation of** ***Nedd9*** **transgenic RARE reporter mice** (a) Genomic structure of the human *Nedd9* gene. The genomic region of the *Nedd9* gene is depicted as a horizontal line. Exons are represented as numbered vertical lines and boxes. Two alternative translational start sites (2A and 2B) are depicted as bent arrows. Shown above is the position of the highly conserved complex RARE characterized in Knutson and Clagett-Dame (2008). Primers, p1 and p2 (depicted by small arrows), are used to monitor DNA quality and to amplify the endogenous *Nedd9* gene. Exons and intronic regions are drawn to scale. (b) Schematic diagram of the human bacterial artificial chromosome BAC, RP11-263D22, used for cloning the targeting constructs used for generation of the transgenic mice. (c) The transgenic targeting constructs contained the region from -5,177 to +15 of the exon 2B promoter (numbering is relative to the exon 2B transcriptional start site +1) with either an intact (top) or modified (bottom, 8 point mutations highlighted) version of the *Nedd9* RARE as characterized in Knutson and Clagett-Dame (2008). A Simian Virus 40 splice donor/slice acceptor (SV40 SD/SA) cassette was added to increase transgene expression followed by the alcohol dehydrogenase translational start codon (ADH-S AUG), *lacZ* coding sequence, and Simian Virus 40 polyadenylation signal (SV40 Poly A). Primers, p3 and p4, are specific to the transgene insert and are depicted as small arrows above the cartoon. Shown are the SphI, BstEII, and SalI sites used for cloning. An SphI/NarI digest was used to linearize and release the targeting fragment from the flanking plasmid sequence. (d) Testing the expression of transgenic plasmids in a cell culture system. *LacZ* reporter gene activity of the pUC19/AUGβgal, *Nedd9*(RARE)-*lacZ*, and *Nedd9*(mutRARE)-*lacZ* plasmids in MCF-7 cells co-transfected with RARβ and RXRβ expression plasmids, and internal transfection control pGL3-Basic then dosed for 24 h with either atRA (10^−6^ M) or vehicle (ethanol). β-gal expression values are normalized to luciferase expression. Values are mean ± standard error. (e) PCR analysis of tail snip DNA from WT and transgenic (Tg) adult male mice. Primers p1 and p2 detect the endogenous *Nedd9* allele, 753 bp. Primers p3 and p4 detect the transgene, 370 bp. (TIFF 344 kb)
