# Supplementary figures and images for: A complex RARE is required for the majority of Nedd9 embryonic expression
Source: Transgenic Res. 2014 Aug 14;24(1):123–34. doi: 10.1007/s11248-014-9825-9 (PMC4274375; doi:10.1007/s11248-014-9825-9)

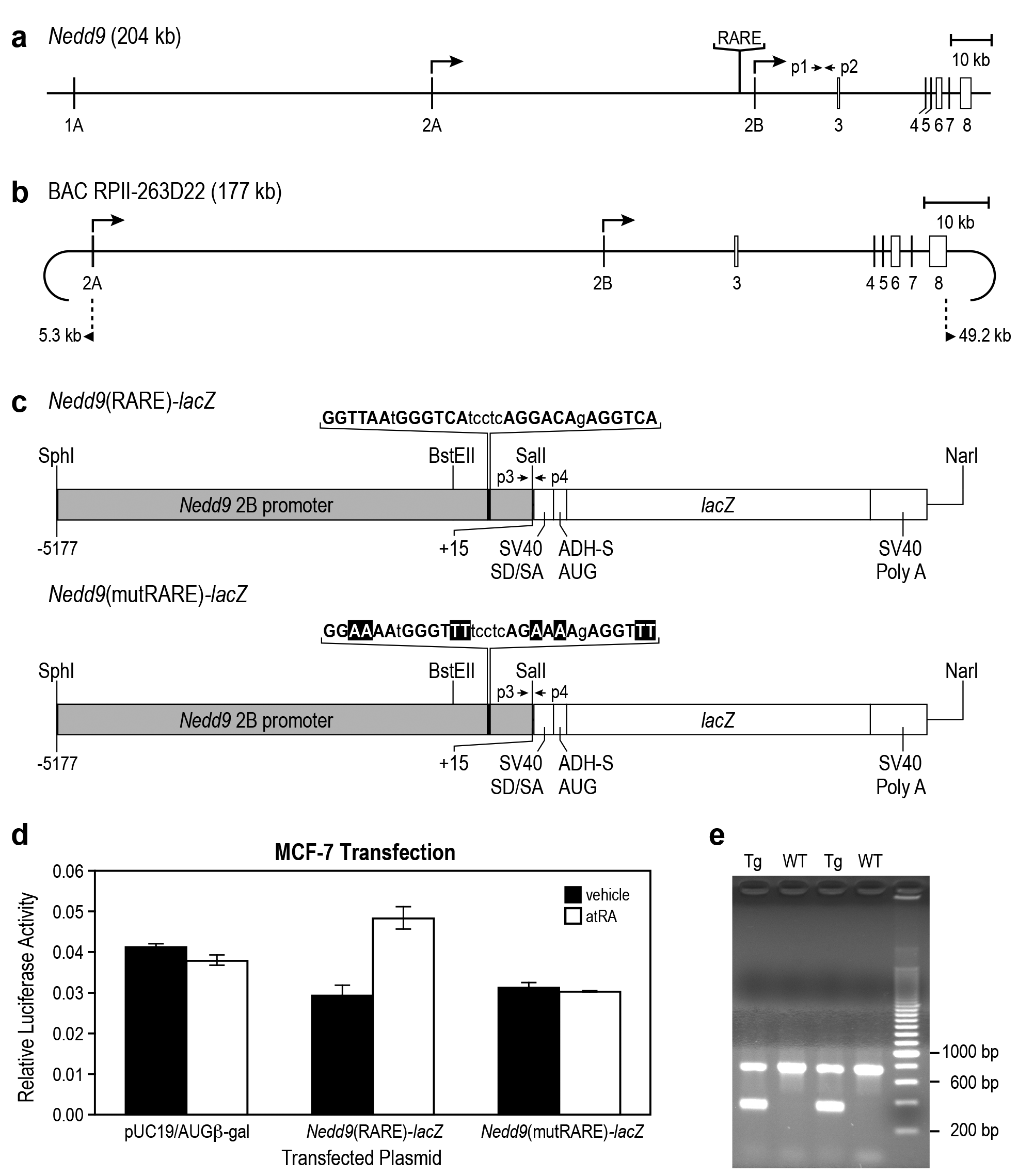

Supplement: Supplementary file 1 — Online Resource 1 Strategy for generation of Nedd9 transgenic RARE reporter mice (a) Genomic structure of the human Nedd9 gene. The genomic region of the Nedd9 gene is depicted as a horizontal line. Exons are represented as numbered vertical lines and boxes. Two alternative translational start sites (2A and 2B) are depicted as bent arrows. Shown above is the position of the highly conserved complex RARE characterized in Knutson and Clagett-Dame (2008). Primers, p1 and p2 (depicted by small arrows), are used to monitor DNA quality and to amplify the endogenous Nedd9 gene. Exons and intronic regions are drawn to scale. (b) Schematic diagram of the human bacterial artificial chromosome BAC, RP11-263D22, used for cloning the targeting constructs used for generation of the transgenic mice. (c) The transgenic targeting constructs contained the region from -5,177 to +15 of the exon 2B promoter (numbering is relative to the exon 2B transcriptional start site +1) with either an intact (top) or modified (bottom, 8 point mutations highlighted) version of the Nedd9 RARE as characterized in Knutson and Clagett-Dame (2008). A Simian Virus 40 splice donor/slice acceptor (SV40 SD/SA) cassette was added to increase transgene expression followed by the alcohol dehydrogenase translational start codon (ADH-S AUG), lacZ coding sequence, and Simian Virus 40 polyadenylation signal (SV40 Poly A). Primers, p3 and p4, are specific to the transgene insert and are depicted as small arrows above the cartoon. Shown are the SphI, BstEII, and SalI sites used for cloning. An SphI/NarI digest was used to linearize and release the targeting fragment from the flanking plasmid sequence. (d) Testing the expression of transgenic plasmids in a cell culture system. LacZ reporter gene activity of the pUC19/AUGβgal, Nedd9(RARE)-lacZ, and Nedd9(mutRARE)-lacZ plasmids in MCF-7 cells co-transfected with RARβ and RXRβ expression plasmids, and internal transfection control pGL3-Basic then dosed for 24 h with eith [file 11248_2014_9825_MOESM1_ESM.tif]
